# Latency and Crisis: Mutual Aid Activism in the Covid-19 Pandemic

**DOI:** 10.1007/s11133-022-09513-7

**Published:** 2022-08-05

**Authors:** Elisabetta Ferrari

**Affiliations:** Department of Sociology, School of Social and Political Sciences, Adam Smith Building, Bute Gardens, G12 8RT Glasgow, UK

**Keywords:** Activism, Mutual aid, Digital ethnography, Instagram, Digital activism, Latency, Mobilization, Crisis, COVID-19, Pandemic, Collective identity

## Abstract

Activists have responded to the Covid-19 pandemic by organizing for mutual aid: creating collective action to meet people’s material needs and build ties of solidarity. I examine the difficulties encountered by mutual aid activists during the pandemic through Alberto Melucci’s notions of latency and collective identity. Through digital ethnographic observations of the Instagram accounts of mutual aid groups based in Philadelphia, USA, as well as interviews with the activists, I explore how mutual aid, conceptualized as latency work, was practiced by activists in the unprecedented conditions of the pandemic and how activists approached collective identity processes. I show that activists experienced a compression of latency and mobilization within the crisis context of the pandemic, which made it more difficult for them to pursue the construction of a collective identity. I also suggest that the effects of this compression were further exacerbated by the logic of immediacy that characterizes social network sites.

In March 2020, with most of Europe under lockdowns and an enormous increase in Covid-19 cases in the United States, a number of grassroots groups began to organize to help those who were hit particularly hard by the pandemic, by cooking food, providing groceries to people in need, raising funds, distributing hygiene products and medicines, facilitating access to resources, and supporting incarcerated individuals. This sudden wave of grassroots activism engendered by the pandemic seized a label that has long been important for social movements, community organizing, and the workers’ movement but rarely enters the mainstream: mutual aid.

Mutual aid can be defined as “collective coordination to meet each other’s needs” (Spade 2020, 7) and “build a shared understanding about why people do not have what they need” (Spade 2020, 9). Mutual aid projects thus simultaneously offer material support to people while working to educate them on the root causes of inequality and getting them involved in political activity. Mutual aid has a long history in the United States, particularly among communities of color. The most well-known example is the work of the Black Panther Party, who created the Free Breakfast program, which provided meals to low-income children, as well as a system of community health clinics (Nelson 2011). Mutual aid serves the dual purpose of improving the material conditions of the community while building ties of solidarity and recruiting people into the struggle for social justice.

The wave of COVID-19 mutual aid activism that we have seen since Spring 2020 is unprecedented in scale and has attracted many people who are new to activism. The physical distancing that characterized the initial phases of the pandemic shaped this process by driving activists to use commercially available digital technologies (from Google Drive to Venmo to Instagram) for most of their work; while offline, low-tech work – preparing food, distributing groceries, packing deliveries – remains crucial to mutual aid, activists have had to rely on digital technologies to communicate with each other, arrange meetings, and make decisions.

This paper focuses on mutual aid groups that have emerged in Philadelphia during the Covid-19 pandemic. Like other cities in the United States, Philadelphia saw an explosive growth of mutual aid efforts in conjunction with nationwide protests for racial justice in the Summer of 2020. I look at these cases of mutual aid activism through the lens of Alberto Melucci’s (1989, 1996) work on social movements. Drawing on a digital ethnographic study of Philadelphia-based mutual aid groups, I use Melucci’s distinction between movement mobilization and latency and his attention to collective identity processes to make sense of the difficulties encountered by these activists. I explain these difficulties as the result of an overlap between latency and mobilization, which, through a metaphor taken from sound mixing, I describe as a compression: a process by which the difference between latency and mobilization is flattened, pushing even phases of latency towards increased visibility. I also suggest that the effects of this compression have been further exacerbated by the logic of immediacy (Barassi 2015) that characterizes social media.

## Mutual Aid and Covid-19

This paper looks at the emergence of mutual aid activism during the Covid-19 pandemic in Philadelphia, which is a poor and profoundly unequal city. While the city is increasingly diverse in its make-up, it remains highly segregated by neighborhood and stratified by poverty levels that vary drastically among different racial and ethnic groups, with 40% of Hispanics and 27% of Black people living below poverty level (The Pew Charitable Trusts 2021). The impact of Covid-19 in Philadelphia was severe – and unequal. According to the Pew Charitable Trusts (2021), “Black and Hispanic residents of Philadelphia were two and three times more likely than White residents, respectively, to lose jobs and income, and to know someone who died from the coronavirus” (1–2).

In 2020, Philadelphia also saw massive mobilizations for racial justice and against police brutality. Like in many other cities in the US, large demonstrations took place in Philadelphia after the murder of George Floyd in Minneapolis in the summer of 2020. The protests in the city were intense, prolonged, and marked by the violent reaction of law enforcement, which included the teargassing of a residential neighborhood in historically Black West Philadelphia (Whelan et al. 2020) and of a group of protesters marching on the highway (Koettl et al. 2020). In October 2020, more protests were organized in response to the killing of Walter Wallace Jr., who was shot by the police in West Philadelphia during a mental health crisis (Hurdle 2020).

It is in this context that Philadelphia-based activists began organizing for mutual aid. While some mutual aid projects (like Mutual Aid Philly and the Philly Socialists’ Mutual Aid support project) started in the early weeks of the pandemic in 2020, many more emerged in subsequent months. By Summer 2020, even mainstream media began covering this wave of mutual aid in the city, focusing in particular on one of its most visible manifestations: community fridges (Scott 2020).

In taking up the label of mutual aid, activists connected their efforts to the long and nonlinear histories that characterize the concept and practice of mutual aid in the United States and beyond. While a review of the different approaches and historical applications of the notion of mutual aid is beyond the scope of this paper, it is important to note that current activists have found an entry point into these debates in recent work by Spade (2020). For Spade (2020), mutual aid projects “directly meet people’s survival needs, and are based on a shared understanding that the conditions in which we are made to live are unjust” (7). Spade identifies three key elements of mutual aid: it responds to people’s survival needs and grounds these responses in an awareness of the systems of injustice that created those needs; it builds solidarity and mobilizes people; it is based on participation and collective action (Spade 2020, 9–20).

In this paper, I conceptualize mutual aid activism through the lens of Melucci’s (1989, 1996) cultural, process-oriented approach to the study of social movements. This approach can help us make sense of the challenges encountered by Covid-19 mutual aid activists, who attempt to build a system of solidarity under unprecedented conditions of crisis, which have also been marked by extraordinary moments of mobilization, like the Black Lives Matter protests of Summer 2020.

## Mutual Aid as Latency

Melucci (1989, 1996) developed a theory of collective action that centered cultural processes. Looking in particular at the “new social movements” that emerged in the 1970s, such as the women’s movement, he made important contributions to the study of collective action. Two are particularly useful to understand mutual aid. First, Melucci criticized the “myopia of the visible” (Melucci 1989, 44) that led social movement scholars to privilege visible action – mobilization – over other processes. He thus introduced the distinction between visibility (or mobilization) and latency, intended as different phases in the life of social movements. Second, he argued that “collective action is not a fact but a process” (Melucci 1989, 45) and that the task of social movement theory is to explain how and why movements come to exist as collective actors; this means especially understanding how collective identity is constructed by movements (Melucci 1996, 70). I use both insights to conceptualize mutual aid and the difficulties encountered by activists.

Distinguishing between mobilization and latency helps us understand processes that are often invisible to observers of social movements and frequently go unnoticed by activists, too. Although it is easier to picture movement work that involves demonstrations and direct action during peak moments of conflict, activism also goes on both before and after these intense moments of mobilization. Melucci argued that movements oscillate between phases of latency and visibility (Melucci 1996, 174). Latency keeps movements going in-between shorter bursts of mobilization: it is when “the potential for resistance or opposition is sewn into the very fabric of daily life” (Melucci 1989, 71). Latency is what makes mobilization possible; it is a movement’s “effective strength” (Melucci 1989, 71). It is during longer periods of latency that activists try to practice lifestyles in accordance with their beliefs through hidden, “submerged” everyday actions and relationships, developing day-to-day practices that embody their ideals outside and beyond moments of mobilization.

I argue that mutual aid *is* latency work. Although it can arise from and feed into bursts of visible, intense, political mobilization, mutual aid is mostly aimed at sustaining networks of care, survival, and solidarity for longer periods of time. Melucci talks of latency phases as nourishing collective action through “the daily production of alternative frameworks of meaning” (Melucci 1989, 70), which activists practice in their day-to-day lives. This daily, almost mundane, prefigurative aspect is clearly evident in mutual aid work, which tries to break free of capitalist norms and internalized attitudes surrounding deservingness, resource sharing, poverty, etc. Mutual aid activists strive to meet people’s basic needs by putting in place alternative arrangements that bypass market relations and center collective care (see The Care Collective 2020); mutual aid offers people the chance to imagine that there might be different ways of existing, outside of white supremacist capitalist structures. To continue with Melucci’s metaphor, mutual aid sews new threads of resistance into the patterns of everyday life.

Further, thinking of mutual aid as latency also helps us make sense of how (relatively) infrequently we talk about mutual aid activism. Melucci’s distinction between phases of mobilization and latency was a response to what he called a “myopia of the visible” (Melucci 1989, 44), which characterized (and to a certain degree, still characterizes) social movement studies: an overwhelming focus on moments of intense mobilization and a neglect of other, less visible – but no less important – processes. Mutual aid activism also falls victim to this neglect. In fact, while we know that mutual aid has long been part of social movements and of marginalized communities’ lives, this has not been a widely explored topic in the social movement literature. But even more interestingly, this relative neglect of mutual aid is also present among activists themselves; as Spade (2020) reminds us, mutual aid is “often a part of movement work that is less visible and less valued” (3); this neglect is likely grounded in the general devaluation of care work due to its gendering (The Care Collective 2020). A focus on latency highlights the importance of mutual aid work despite its reduced visibility, by decentering the importance of visible forms of mobilization and embracing a more cyclical view of activism.

Considering mutual aid work as a form of latency can also highlight the specific tensions encountered by mutual aid activists in the Covid-19 pandemic. In fact, because mutual aid projects emerged all over the world in conjunction with and in response to a truly unprecedented global crisis, activists found themselves doing latency work under such heightened conditions of crisis. They were also incredibly visible, in ways that, as explained, are not typical for mutual aid work: from mainstream media coverage to social network sites, it seemed like mutual aid was suddenly on everyone’s mind. Interrogating the relationship between these mutual aid projects and the crisis conditions in which they emerged can help us understand the tensions that activists had to navigate.

## Collective Identities in the Covid-19 Pandemic

Conceptualizing Covid-19 mutual aid activism through Melucci’s (1989, 1996) scholarship foregrounds not only the relationship between latency and visibility but also the importance of considering processes of collective identity formation as central to movement work. For Melucci, collective identity is an open-ended and contradictory process, based on constant negotiations within movements (Melucci 1996, 70): “collective identity is an interactive and shared definition produced by several interacting individuals who are concerned with the orientations of their action as well as the field of opportunities and constraints in which their action takes place” (Melucci 1989, 34). Crucially, it is this process of collective identity construction that allows for the formation of social movements (Melucci 1996, 75): people do not act collectively simply because they share a similar concern or value, but rather it is only through an interaction, through a collective process, that actors can come to recognize a concern, value or injustice as such (Melucci 1989, 193).

For activists that became involved in mutual aid work during the Covid-19 pandemic, any collective identity formation processes will likely have taken place through digital technologies such as Zoom, Slack, or social network sites. Much has been written about collective identity and digital technologies, including by scholars who embrace a Meluccian approach to social movements. They have highlighted how digital media can become a locus for the construction of collective identities, but that this process is difficult, contradictory, and marked by power differentials. Drawing on an analysis of the Occupy movement, Kavada (2015) argued that communication processes taking place through social media *can* create a collective actor, despite the many difficulties of using these platforms for activist aims. Some of these difficulties have to do with the individual-centric logic of social media and how that clashes with decentralized political processes, such as those employed by Occupy (Ferrari 2016). Some have to do with the “politics of visibility” that is encouraged by social media, which redefines how identity is constructed by movements (Milan 2015). And some are derived by the “temporality of immediacy” that characterizes social media, and that pushes activists to try to keep up with a constant flow of information, while making “processes of political elaboration,” which are typically time-consuming, more difficult (Barassi 2015; Kaun 2016 for a historical comparison). These difficulties do not mean that collective identity processes do not exist in contemporary social movements, but they are often happening outside of news feeds and hashtagged tweets. For instance, Treré (2015) showed that collective identity is bolstered by digitally mediated internal communication practices through group chats and direct messages – the “backstage” of activist communication; by focusing only on the “frontstage”, i.e., external communication on social media, activists risk neglecting these important invisible processes.

If collective identity processes mediated by social media are already always difficult for activists, they are likely to be even more complicated for mutual aid activists in the context of emergency generated by the pandemic. This paper investigates the collective identity processes of mutual aid activists in Philadelphia and understands them through the interplay of latency and visibility.

## Research Questions and Methods

This paper examines the following questions: How is mutual aid, considered as latency work, carried out by Philadelphia-based activists during the Covid-19 pandemic? How do these mutual aid activists construct a sense of collective identity during the Covid-19 pandemic?

I address these questions through a digital ethnographic approach. I began keeping track of various mutual aid efforts in Philadelphia as they were developing in the Spring of 2020; given my previous research on Philadelphia-based radical movements, I was able to follow the emergence of these projects and their connections to the city’s activist scene. While I did not set out to specifically observe this work on Instagram, over time I found that it was this platform where mutual aid activists were most active. I followed the Instagram accounts of the various groups as they were emerging; I sometimes found out about new projects through media coverage but mostly by following the actors I was already studying, looking up the projects they were tagging or engaging with, and taking advantage of Instagram’s algorithmic-driven recommendations (Leaver et al. 2020). I took screenshots of the content posted by these accounts and annotated them. I took more systematic fieldnotes and screenshots about what I was observing for the month of May 2021, paying particular attention to Stories and the circulation of content across different accounts.

My ethnographic work identified 19 accounts, which can be seen in Table 1. I decided to limit my analysis to the (public) accounts of different mutual aid projects, not of individuals engaged in mutual aid. This decision was informed by what I observed on Instagram: while individual activists were sometimes tagged by the groups’ accounts, mutual aid projects mostly communicated with each other through the official group pages.^1^

**Table 1:** Mutual aid groups followed on Instagram, their number of followers, and their main activity. Activists from groups indicated with * were interviewed. The follower count is current as of October 6th, 2021

Name	Follower count	Main activity
Citywide Mutual Aid	3,665	Donations collection
Community Action Relief Project (CARP)*	1,488	Food distribution and/or delivery
Coral Street Fridge*	497	Community fridge
Funds Y’all	1,774	Direct aid
Germantown Community Fridge*	4,004	Community fridge
Homies helping homies	4,474	Food distribution and/or delivery
Mobile Donations Collective	395	Donations collection
Mutual Aid Philly*	3,865	Food distribution and/or delivery
Northwest Mutual Aid Collective*	290	Food distribution and/or delivery
Papermill Food Hub*	779	Food distribution and/or delivery
People’s Kitchen	3,483	Food distribution and/or delivery
Philly Survival Collective*	119	Food distribution and/or delivery
PHL Laundry Support	980	Donations collection
Powelton Community Fridge	397	Community fridge
South Philadelphia Community Fridge	6,417	Community fridge
Spring Garden Community Pantry	168	Community fridge
The Family Fridge*	848	Community fridge
The People’s Fridge (Fridge on 52nd )	7,351	Community fridge
West Philly Bunny Hop	6,552	Food distribution and/or delivery

I initially used my personal Instagram account to follow the groups, but then created an ad-hoc research account to engage more directly with the groups. This research account (@mutualaidresearch) clearly stated my name, affiliation, and the fact that I was conducting a study on mutual aid. A link in my Instagram bio directed users to a website with a detailed explanation of the study, including informed consent materials.

**Table 2:** List of interviewees, with pseudonym, pronouns, and age at the time of the interview

Pseudonym	Pronouns	Age
Amanda	She/her	30
Ashley	She/her	30
Eric	He/him	18
Jayden	He/him	30
Jessica	She/her	26
Joshua	He/him	35
Julian	He/they	24
Mary	She/her	30
Patricia	She/her	34
Samuel	He/him	52
Sarah	She/her	29

I also conducted semi-structured interviews with 11 activists involved in 7 of the mutual aid projects considered (see Table 2). I recruited interviewees through Instagram, both by sending a direct message to the accounts and by posting recruitment materials through my research account. I also recruited interviewees by emailing the publicly available emails of the groups. The interviewees received an honorarium of $25 for their participation. The interviews were conducted on Zoom and then transcribed by a professional service.

The interview guide was informed by my digital ethnographic observations of the mutual aid groups and focused on different topics: interviewees’ involvement with the mutual aid project and previous experiences of activism, the functioning of the mutual aid project, the political meaning of mutual aid, the use of digital technology in the mutual aid project, and the role of Instagram.

The interviewees are all identified with a pseudonym. While I sought the interviewees’ consent to utilize the names of the mutual aid projects for this work, I chose not to associate interviewees’ pseudonyms to the names of their projects, in order to prevent members of the (relatively small) Philadelphia mutual aid community from recognizing each other.

The fieldnotes and the transcripts of the interviews were analyzed together, through thematic coding (Braun and Clarke 2006). By bringing together an ethnographic exploration of their communication on Instagram with in-depth interviews with activists, I was able to consider both “frontstage” and “backstage” processes (Treré 2015)^2^.

## Routines and Alternatives

Messaging one another on Slack or via texts, looking for a driver that can deliver items from point A to point B, asking for and receiving donations on Venmo, and sharing an Instagram story about the most recent accomplishment or the most urgent activity that they need help with: this is the day-to-day of mutual aid activists. Despite the great variety of activities carried out by the different projects, activists’ mutual aid labor is similar across the board. It often looks very mundane; it might overlap with tasks that activists would normally do at work (emailing) or in their homes (cooking or cleaning a fridge).

Activists have used Instagram consistently to show this labor, which is prominently displayed by the accounts I followed in the form of Stories and posts, pictures of filled fridges and distribution tables, videos of volunteers packing supplies, and even infographics about how funds were collected and spent. As Eric detailed:

Yeah, Instagram is basically our best, it’s our main platform. Everything is run through here. We provide our daily fridge checks ... Not daily, I think once every two days, once every three days, we just send a video of what’s inside the fridge. Use Instagram if we need a driver to pick up donated goods, we just put out a message on our story.

Like many other activists, Eric described Instagram as an all-encompassing platform, which can be used to “run” the mutual aid project (in this case, a community fridge), particularly when it comes to communicating with potential donors, potential volunteers and people who might benefit from accessing the fridge. For Eric and other activists, Instagram showcases the labor that goes into running the mutual aid projects.

I suggest that the most important function of this Instagram content is to routinize the idea of mutual aid, in an effort to make it sustainable. By that I mean that this activist use of Instagram can help show people that mutual aid can be part of their daily lives with relatively little effort – that it can become a routine. This content informs users of the platform about different ways to support the mutual aid groups and emphasizes actions that have a low barrier for entry – Venmo-ing a few dollars, bringing canned food to a pantry. This routinization of mutual aid seems particularly important given how little mutual aid was talked about before the Covid-19 pandemic. In contrast to other well established activist practices – from protest rallies to hashtag campaigns – mutual aid work is much less familiar to the general public. This kind of content thus educates the public about what mutual aid is and presents it as activist work that can be integrated into people’s daily lives. For instance, a series of graphics contributed by a volunteer of the People’s Fridge explained to people that “mutual aid has a history in Black, Indigeneous and Brown communities,” that “it is important to honor that work by uplifting the voices and concerns of BIPOC” and that “mutual aid is about treating or neighbors and community with dignity and respect” (@thefridgeon52nd 2021).

A lot of this Instagram content is devoted to explaining the rules of the different mutual aid groups, i.e., what people should and should not do. Many of the groups display these rules in a prominent position on their Instagram accounts, using the Highlights function, where they save otherwise temporary content from their Stories. For instance, the Germantown Fridge created a video with a set of instructions on how to donate ready-made meals to the fridge; in the video, a volunteer shows properly packaged and labelled food while captions and a voice-over explain that people should “make sure you label food with ingredients, date it’s made & when it should be eaten by” (@germantownfridge 2021). The People’s Fridge has a Highlight called “Donations ?!?,” which hosts content about best practices related to donating. One of the panels displays a photo of food in a plastic bag, hanging outside the fridge, captioned as:

Hey, this is not helpful. Please take your donations out of the bag and stock them in their respective baskets! There are countless posts and stories and highlights on how to donate. Here is how to donate – take your donations out of the boxes and bags they’re in and place them inside the fridge. Produce should be individually bagged and labeled. If they are not fridge items or essentials, please redistribute them to fridges that take those items. (@thefridgeon52nd 2020)

This focus on educating people on how to correctly contribute to the mutual aid projects is central to the content posted by the different groups on Instagram. It tries to convey the sense that there are clear and (relatively) effortless actions that regular people can perform to participate in mutual aid on an ongoing, if not daily, basis. By working to make mutual aid intelligible and mundane, mutual aid activists show the public how to think differently about life under capitalism; they embed a critique of the system within actions that rewrite the scripts of daily life, showing alternative ways to think about people’s basic needs. This is the work of latency: nurturing the “production of alternative frameworks of meaning” (Melucci 1989, 71) that can be put in practice by people in their day-to-day.

## Latency and Emergencies

This routinization of mutual aid activism stands in sharp contrast with the clear attention to events and emergencies that is also in full display in these accounts. When international – such as the Covid-19 crisis in India in April 2021 – or domestic emergencies occur, mutual aid activists use Instagram to draw attention to the issue. This typically takes the form of content that is either created by the activists or reshared from other accounts, and that informs their followers about the event at hand. But this content also often tries to provide avenues for people to get involved with solidarity efforts, especially those connected to mutual aid ideals. For instance, when conflict broke out between Israel and Hamas in May 2021, many mutual aid groups produced and reshared content about Israeli-Palestinian relations and the history of the conflict; they took part in and documented the pro-Palestine marches in Philadelphia, and reposted calls for donations towards Palestinian solidarity groups, such as the Philadelphia Free Palestine Coalition. During Pride month, the accounts devoted significant attention to specific pro-LGBTQI + fundraisers and solidarity events. When severe winter storms hit Texas in February 2021 and the state’s power grid collapsed, accounts encouraged donations to Texas-based mutual aid groups that were helping people through the outages. And perhaps even more evidently, during the protests following the killing of George Floyd in the Summer, and of Walter Wallace Jr. in the Fall of 2020, the Instagram accounts of the mutual aid groups shared calls for action, information on how to protest safely (e.g., primers on digital security for protesters) and documented their involvement in the protests. They also oriented their work towards supporting protesters, including jail support. For instance, the Community Action Relief Project (CARP) coordinated the packing and drop-off of “supply bags,” plastic bags containing water or other soft drinks, snacks, first aid items, masks, sanitizer, and an information sheet with protest tips, which they handed out to protesters. One of CARP’s activists explained:

Last year especially in the wake of all of the protests that erupted last summer, CARP was transformed into a hub for resources to bring to protest support, jail support. So there were a lot of ways in which it could be repurposed to support that kind of action. And at the time I remember we were having conversations about like, “Well, this is what’s important right this second. Right now we get water to the people who are on the streets.”

For this CARP activist, it was crucial for the mutual aid project to be flexible and able to respond to the extraordinary mobilization in Philadelphia: what was important at that very moment. Of course, not all events or emergencies reoriented the work of the mutual aid groups in the same way; to the best of my knowledge, for instance, none of the groups changed their practice to respond to the power outages in Texas or the Covid-19 crisis in India. But they used Instagram to draw attention to those events and emergencies; they posted intensively about that particular issue for a limited amount of time.

The alternation of routines and emergencies, day-to-day actions, and extraordinary mobilizations marks the way in which the mutual aid projects communicate with their followers on Instagram. This is part of larger issues that have affected the mutual aid projects and that I argue should be understood as resulting from the contradictions of doing latency work under conditions of crisis. Movements are always sensible to ebbs and flows in attention and participation; activists’ personal lives often shape the work of movements, coalitions can break down and dissolve, and activist burnout is a real concern. Yet these mutual aid projects, because they emerged at the height of an unprecedented crisis, have been particularly exposed to the turmoil of 2020–2021: these conditions of crisis have been an enabler, but also a constraint, for these mutual aid activists. Many interviewees described how pandemic-related job loss and/or work-from-home arrangements gave them more time to get involved in activism. At the same time, the fact that the pandemic intensified conditions of hardship and injustice also made interviewees more willing to step up. But if this increased biographical availability (McAdam 1986) facilitated the emergence of mutual aid projects in early 2020, the post-vaccine attempts to resume in-person economic and social activities in 2021 left the activists with fewer people interested in participating in mutual aid and less attention towards their efforts. While the number of people who benefit from mutual aid projects is still significant, interviewees report having a reduced number of drivers for grocery deliveries, less success in fundraising, and even finding that some public assistance programs have ceased operations. Some of the projects have had to scale back their activities and all of them are thinking about how to be more sustainable in the long run. As mentioned, ebbs and flows are common in the life of social movements, but these groups have experienced all of these changes during a relatively short time span, punctuated by major external events.

The intense protests for racial justice of Summer 2020 were a particularly salient event, which shaped, in different ways, the trajectory of mutual aid groups in the city. For one activist, the protests were the main motivation for getting involved in mutual aid, after being among the protesters teargassed and arrested by the police on the highway (Koettl et al. 2020): “I wasn’t even a protester in general before last year. But then I started doing it. And then I was arrested on [Interstate] 676 when they were teargassing us and stuff. And so I was like, ‘I’ll do this every day’” (Patricia). Groups that were already up and running found themselves adjusting their work to this extraordinary mobilization. Mutual Aid Philly, for instance, started receiving a high volume of monetary donations during the protests, which made the organizers highly uncomfortable; as a group that is composed mostly of white activists and serving a predominantly white and Latinx population in South Philadelphia, Mutual Aid Philly activists felt “in a really weird position (…) of redistributing all of this money,” presumably donated in support of Black communities. For CARP activists, the protests even prompted a rethinking of the set-up of the mutual aid project itself, which ultimately led to an entirely different organizational structure – “there was the George Floyd uprisings which changed the landscape and we totally shifted gears.” Even if these mutual aid activists were not necessarily all involved in the protests for racial justice, that mobilization profoundly shaped their activism: by recruiting new people into mutual aid and providing unexpected financial donations but also by reshaping the kind of work carried out by these groups and rendering it increasingly visible to the public and to the media.

If we look at these processes through Melucci’s work, we can see that mutual aid activists experienced a clash between latency and visibility; their daily latent work of mutual aid was not embedded into ordinary routines and processes but rather coexisted with times of extraordinary events and emergencies. As we will see below, this compression of latency and visibility also marked the collective identity processes of this movement.

## Searching for a “Sense of We”

On Instagram, the different mutual aid projects often engage with each other using the affordances of the platform: they repost each other’s posts and Stories, they tag each other, they like posts and add supportive comments. Each account conveys the sense that they are part of a broader field of mutual aid work in Philadelphia; they don’t try to portray themselves as the only entity that’s doing mutual aid (or doing it in the right way) but rather try to link to other groups. As Julian explained:

I also try to focus on sharing other resources, sharing other organizations. Just so that, one, you can get people to refer to other organizations, other ways that they can get help. But also building a relationship with other organizations, so that they can also promote us as well.

Using their Instagram accounts to tag other accounts and “promote” other groups is thus both about helping people find the support they need and trying to connect with other mutual aid projects (or other organizations). For instance, I have observed that different accounts have shared a map which lists several community fridges in Philadelphia in their Stories (e.g., @southphlcommunityfridge 2021); in general, the accounts are very intentional in amplifying each other’s fundraising requests, resharing informational materials and generally acknowledging each other through tags, likes and comments.

The affordances of Instagram have also been useful to establish forms of coordination between the mutual aid projects. In particular, various community fridges in Philadelphia created a group chat via Instagram, which they use to exchange information and connect with each other. Several of the activists mentioned the community fridge group chat and described it very positively. Jessica described the chat as “a little mix of everything”: “We’ll troubleshoot stuff with each other, share graphics, and share info about certain give-aways.” Through this group chat, fridge activists “send food back and forth to each other” (Joshua), help each other if the fridges break down and boost each other’s posts about special events, fundraisers, etc. (Jessica).

There have also been more explicit and sustained coalition-building efforts among the different groups. Philadelphia City Councilmember Kendra Brooks, for instance, gathered different mutual aid groups in early 2021 to spark a conversation about community care in the city. This virtual gathering gave rise to a “city wide mutual aid resource exchange,” as Mary described it. This resource exchange is supposed to help different groups learn from and support one another. At the time of the interviews, the groups had met a couple of times in 2021 and were planning another gathering. Amanda explained:

We’ve been talking about coalition-building. That’s how I met the folks from Mutual Aid Philly. They were trying to build a resource exchange type of deal where if one group has a surplus of one thing that somebody else needs, maybe we can help each other out in that way. We actually have a mutual aid happy hour scheduled for next month, trying to get as many people from different groups as possible without it being too overwhelming. Share stories and talk about what we’ve all been doing for the last year and a half, because I’m sure we’ve all run into a lot of the same problems and a lot of the same successes, too.

The resource exchange thus tries to facilitate collaboration and information sharing.

Given these evident attempts to connect the different mutual aid projects, can we talk about these groups as being engaged in the process of becoming a collective? Can we see collective identity processes at play within and across these Philadelphia-based mutual aid groups? It is unclear. Both the Instagram group chat and the resource exchange clearly signal a willingness by the activists to connect with each other. But this connection does not seem to take the shape of an ongoing effort to construct a “sense of we” (Melucci 1989, 65). Indeed, the activists are even reluctant to define these efforts as coalition-building, preferring to call them resource exchange (or a “group chat.”).

This does not mean that these coordination efforts are meaningless or unhelpful. Indeed, some of the activists have found a tremendous amount of support from activists in other mutual aid projects. Samuel said that it bothers him if other mutual aid groups are “kind of struggling”: “I will pick up the phone and say, ‘Hey, listen, I just saw your post. Are you okay? Do you need help?’ I think that’s really in the traditional spirit of mutual aid” (Samuel). For Joshua, these relationships have morphed into friendships: “Truthfully it’s turned out that some of my mutual aid friends and people are really showing up a lot more than most other people in my life. They text me like, ‘How are you doing? Are you tired?’” In this sense, the coordination among different mutual aid groups seems to also have given rise to supportive personal relationships.

Overall, however, activists have had an easier time forming strong bonds within the individual mutual aid groups, whether through frequent Zoom meetings about the direction to be taken by the groups, in-person political education (e.g., CARP), or by building connections through the mundane work of mutual aid itself. They found it much harder to create a shared sense of solidarity across different projects. As Ashley put it: “It’s been challenging to connect with other groups and I’m not really sure exactly why that is. This has been an ongoing battle.” Both Patricia and Mary elaborated on this difficulty of forging bonds of solidarity across different groups:

I mean, I feel a sense of comradery with my drivers and my volunteers and these people that come every single week to do these deliveries. And it’s been an incredible experience to be in [my group] but not with other mutual aid groups, no. (Patricia)

I feel like when I’m with people from [my group], it’s like being with my community and my people. It’s one of the most comfortable spaces for me to be in. And I’m friends, I’ve become friends with a lot of the other organizers, just because we’ve met through [my group]. With those other groups, I think part of it is just not totally... One, it’s so hard to build community over Zoom. And I don’t know that we’ve figured out how to do that, or that we’ve... It’s interesting that you raised that because I think we went immediately towards the resource sharing and exchange and maybe we didn’t really figure out, well, who is everyone here and why are you in this work? And what’s your personal stake in this? Which is… I’m now embarrassed to say this, because that’s organizing 101. (Mary)

Understanding other activists’ motivations and stakes – what Mary called “organizing 101” – is a crucial part of collective identity processes. The fact that these kinds of collective discussions have not been part of the meetings of the resource exchange is indicative of a general difficulty of doing collective identity work. When I discussed this issue with the interviewees, they didn’t really know why they found these identity-building processes so difficult. Mary was particularly interested in talking about these difficulties, which puzzled her greatly: “People are just doing their own thing. And I don’t know what will get people excited or interested in collaborating. That for me has been really frustrating. I feel like it’s a code that I haven’t been able to crack.”

Part of the difficulty of developing a common “sense of we” also stems from the different approaches to mutual aid organizing that are deployed by the different groups. Activists are very careful in their critiques of other groups, but it is clear that the differences are profound, for instance in terms of organizational structure (led by charismatic leaders vs. run through consensus), communication style, and political commitment (more or less radical). Several activists remarked on how different the mutual aid groups were from each other. Patricia said that, while the groups connected with each other and “like[d] each other’s posts and stuff,” they “are all doing different things.” These different critiques that activists (politely) formulated speak to widely diverging approaches to mutual aid, which make the mutual aid scene in Philadelphia highly heterogeneous.

Heterogeneity in itself is not incompatible with collective identity formation processes, but these activists have so far not been able to find a way to become a collective actor. Ashley offered her explanation: “It could just be that everybody is so overworked and there’s just so much energy going into these projects and resources that nobody has the time or the energy to build coalitions.” Many other activists talked about experiencing burnout and the toll that mutual aid activism has taken on them during the pandemic. I suggest that these activists’ difficulty in forming a collective identity should be read as the result of a compression between latency and mobilization due to the interlocking crises of the pandemic. What Ashley describes as overwork and lack of time and energy is a result of this compression of modalities: these activists have experienced latency and visibility at the same time, and this has made it more difficult for them to make sense of their activism. As I discuss below, this compression has also been exacerbated by the logic of immediacy that characterizes social media, which encourages activists to adapt to the fast flow of social media communication.

## A Compression of Latency and Mobilization

My analysis highlighted two difficulties encountered by Philadelphia-based mutual aid activist: first, the constant push and pull between the attempt to establish and routinize alternative frameworks and the attention to the events and frequent emergencies that have characterized the course of the Covid-19 pandemic; second, the complexity of putting in place processes of collective identity formation. I argue that both of these difficulties can be explained as the result of a compression of latency and mobilization.

Melucci thought of latency and mobilization as two correlated poles that support each other: while mobilization bolsters solidarity and helps recruit people, latency creates meaning (Melucci 1996). But this is not what mutual aid activists experienced. Because of the extraordinary crisis conditions of the pandemic, activists found themselves trying to build alternative frameworks of meaning for everyday life, while “everyday life” was being constantly transformed before their eyes. They built activist practices that aimed at intervening in longstanding systems of injustice while also trying to focus on how these injustices manifested under emergency conditions. They organized alongside the unprecedented mobilizations for racial justice: the protests inspired them but also changed their work. And they did all this while receiving a tremendous amount of media coverage, social media attention, and donations. Despite their focus on less visible, more mundane forms of activism, especially compared to the intensity of the protests, they became a recognizable force in the social justice-oriented responses to the crises of the pandemic. These conditions of crisis, however, profoundly affected their work by blurring the lines between latency and mobilization.

In the music industry, compression (or dynamic range compression) is a process whereby sound is mixed to reduce the difference between loud and quiet parts; this compression makes quiet sound louder, flattening all sounds towards the louder end. Compression gives listeners a sound that is more homogeneous, but flatter, deprived of the disruptive power that the effective interplay of quiet and loud can give to music. This is the kind of compression that we are observing in the case of mutual aid activists: quiet phases of latency are pushed towards loudness, towards mobilization and visibility.

I argue that when latency and mobilization collide, they do not reinforce, but rather undermine each other. The compression of latency and mobilization makes everything more complicated: it hinders meaning-making processes because it pushes conversations about identity down the line, in favor of attending to whatever is perceived as (and might very well be) a more urgent task. After all, it is exceedingly difficult to focus on creating sustainable structures or alternative frameworks when fighting for survival. But by decentering collective identity processes, this compression can lead to activist burnout, by overwhelming activists with the mechanics of collective action and downplaying the meaning of their work. To come back to the sonic metaphor, dynamic range compression results in music tracks that are constantly loud. It removes depth from the sound and reduces the disruptive power that comes from the contrast of soft and loud sounds. In activist terms, compression weakens movements, because it deprives them of the slow and deep work of latency.

I also argue that the compression of latency and mobilization is further exacerbated by the logic of immediacy (Barassi 2015) that activists encounter in their extensive use of Instagram. Immediacy results from broader shift in informational capitalism and it is prominently engrained in social network sites. Immediacy is problematic for activists because it “challenges processes of political elaboration” (Barassi 2015, 83) by pressuring activists to produce quicker, shorter, carefully timed content that can be visualized and reshared on social media (see also Milan 2015). One activist, Ashley, addressed this by saying that she felt her group was “bad” at social media; she explained that the collective felt they had to post to social media (“I mean, we understand that you have to have social media, I think, to exist, to be considered an entity in this world”), but found it challenging because most of their work was comprised of activities, such as meetings, that would not look good in a post:

I think it makes more sense when you’re doing in-person work because you have images, you have things that are interesting to look at. And when you have remote work, it’s boring. It’s Zoom meetings. What are you going to put on social media? (Ashley)

In the case of the mutual aid groups considered in this paper, the logic of immediacy reinforces the compression of latency and mobilization by encouraging activists to focus extensively on “instagrammable” frontstage communication, as Ashley illustrates. Immediacy thus compounds the already problematic effects of the compression, further pressuring activists to privilege more visible aspects of their work and leaving less space and time for backstage conversation. This is evident, for instance, in the contrast between the Instagram content of the groups, which prominently displays markers of connection among groups (reshared posts, tags) and the tentative attempts at collective identity formation between the activists.

As shown in this paper, social network sites can support activists’ latency work by helping them embed their ideals into the mundane aspects of daily life and deepen their ties of solidarity. However, this is not what these platforms are engineered to accomplish. Having to conform to the logic of immediacy further takes activists away from the difficult task of building a collective, thus exacerbating the problems generated by the compression of latency and mobilization.

## Conclusions

By conceptualizing mutual aid as latency, this paper contributes to our theoretical understanding of the role of mutual aid within social movements and its renewed importance for US-based radical activists. It also shows how crisis conditions can give rise to a compression of latency and mobilization, which pushes activists towards increased visibility, and which is in turn heightened by the logic of digital immediacy; this compression makes it more difficult for activists to organize effectively and nurture solidarity. This conceptualization might prove useful to examine the impact of different types of crises on future social movement organizing beyond the Covid-19 pandemic.

Further, this paper offered insights into the use of Instagram by US-based mutual aid activists and radical activists in general. Although Instagram has become a significant platform for social movements, it has been so far underexplored by activism scholars. Given the longstanding centrality of Facebook and Twitter to both activist organizing and academic scholarship on activism, further studies of Instagram as a platform for radical activism should explore how activists shifted to this platform and how they think about Instagram as a space for social justice activism.

Through its digital ethnographic approach, this paper also contributes to ongoing efforts to understand the complex interplay of social movement processes and digital technologies. By pushing so many social interactions towards online spaces, the Covid-19 pandemic made even more evident what has already been argued by scholars of activism and media: how much digital technologies underpin, and thus influence, the life of contemporary social movements. Digital ethnography is therefore not just crucial to understanding how and why activists might use Instagram or Twitter; it is now indispensable to analyzing how social movements work *in general*, how they become collective actors, and how they make sense of themselves. These collective processes are now so intertwined with digital technologies that our research requires digital ethnographic methods to make sense of such processes and of the challenges faced by activists.

## Footnotes

1 While the interactions I observed were all *public*, i.e., observable without passwords, I chose to draw a distinction between permanent and ephemeral Instagram content: I do not employ screenshots or verbatim transcripts for Stories that I have captured through screenshots or notes, because I consider them ephemeral material that the authors might not want preserved, but I do include verbatim quotes from posts and Stories that have been archived by the accounts, as I consider them to be publicly displayed.

2 In an ideal situation, my research would have also engaged the mutual aid activists as they carried out their activities; I would have chatted with them as they stocked the community fridges or carried out deliveries. But this research, just like this wave of mutual aid activism, took place in difficult conditions, marked by the pandemic and its uncertainties. When I started following these activist groups, I made the difficult choice to not study their work in person, because I did not feel comfortable with taking health risks. Later, as I moved away from Philadelphia for professional reasons, I decided to keep researching these groups from afar, because of my knowledge and connection to the city’s activist scene; but that meant that, even as the pandemic conditions eased, I was not able to engage with this mutual aid work in person. My digital-only engagement, however, mirrors how many activists participated in mutual aid, especially during the earlier phases of the pandemic; I thus approach this analysis by keeping in mind the heightened importance of digital spaces for both research and activism.
